# Supplementary material for: Genomic Analysis of Aspergillus Section Terrei Reveals a High Potential in Secondary Metabolite Production and Plant Biomass Degradation
Source: J Fungi (Basel). 2024 Jul 22;10(7):507. doi: 10.3390/jof10070507 (PMC11278011; doi:10.3390/jof10070507)
Supplement: Supplementary file 1 [file jof-10-00507-s001.zip › Figure S1.pdf]

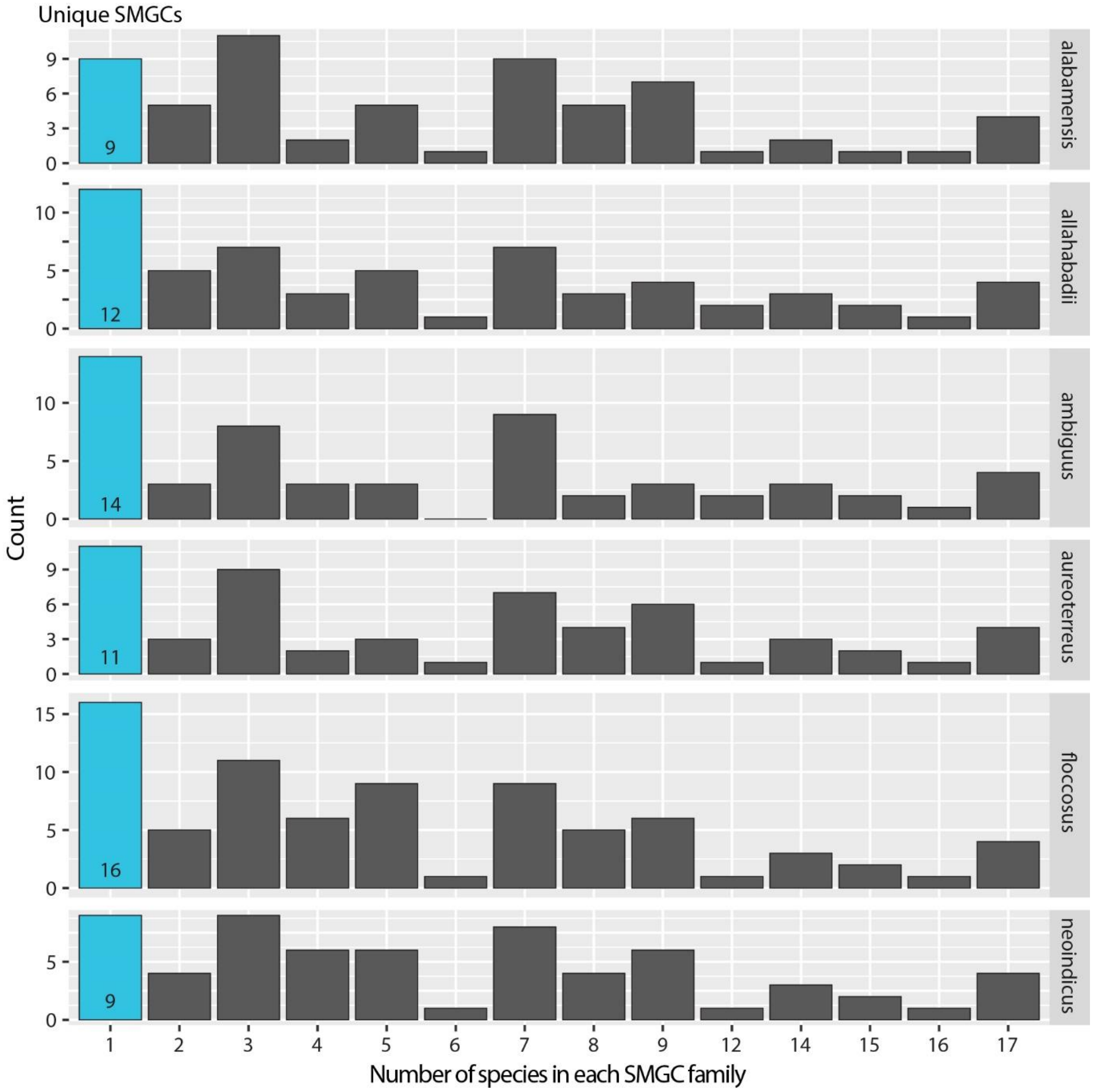

Figure S1 - Overview of SMGC family size from all species in section *Terrei*. Each column shows the number of SMGC families that is either unique to the species or is shared with 1-26 other species tested in this study.
