# Supplementary material for: Genomic Analysis of Aspergillus Section Terrei Reveals a High Potential in Secondary Metabolite Production and Plant Biomass Degradation
Source: J Fungi (Basel). 2024 Jul 22;10(7):507. doi: 10.3390/jof10070507 (PMC11278011; doi:10.3390/jof10070507)
Supplement: Supplementary file 1 [file jof-10-00507-s001.zip › Figure S2.pdf]

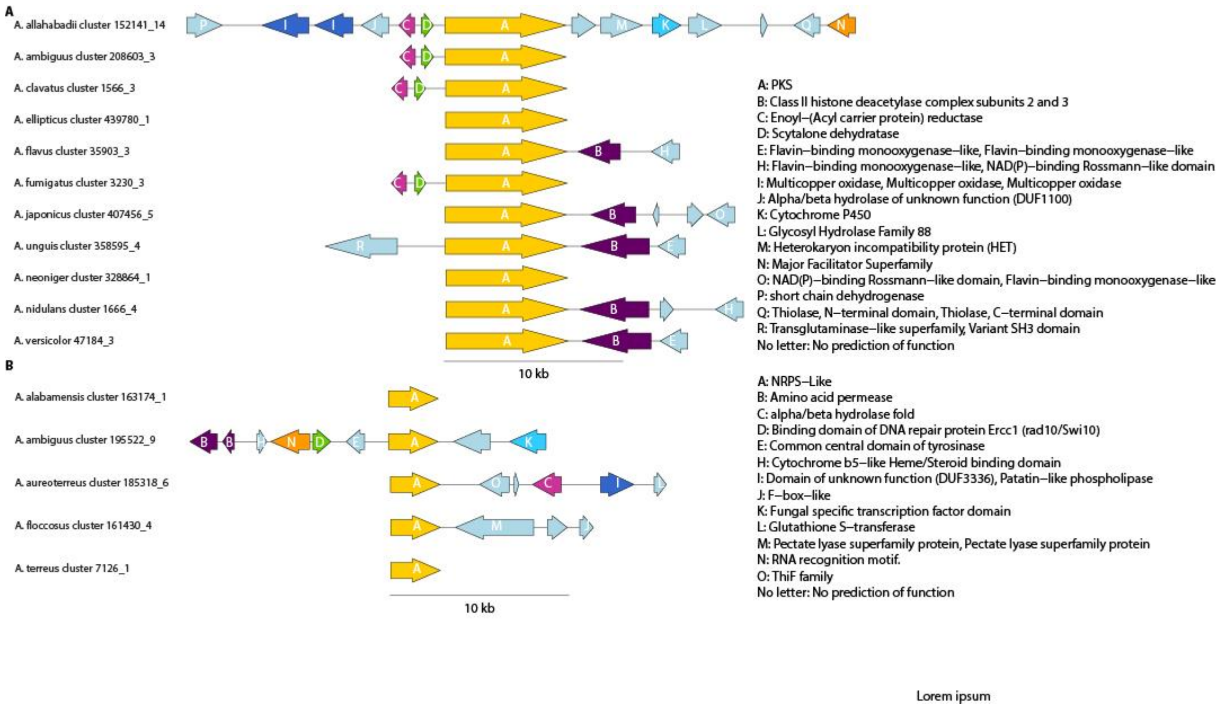

Figure S2 - Synteny maps of SMGC families for pigment clusters. A - Clusters predicted with homology to the *A. nidulans wA* and *A. fumigatus pksP* gene. **B** - Clusters predicted with homology to the Asp-melanin biosynthesis pathway including the aspulvinone E synthase gene *melA* from *A. terreus.* Note that the clusters presented are the raw predictions. Cluster names refer to their naming in Suppl. Table 2.
