# Supplementary material for: Genomic Analysis of Aspergillus Section Terrei Reveals a High Potential in Secondary Metabolite Production and Plant Biomass Degradation
Source: J Fungi (Basel). 2024 Jul 22;10(7):507. doi: 10.3390/jof10070507 (PMC11278011; doi:10.3390/jof10070507)
Supplement: Supplementary file 1 [file jof-10-00507-s001.zip › Figure S3.pdf]

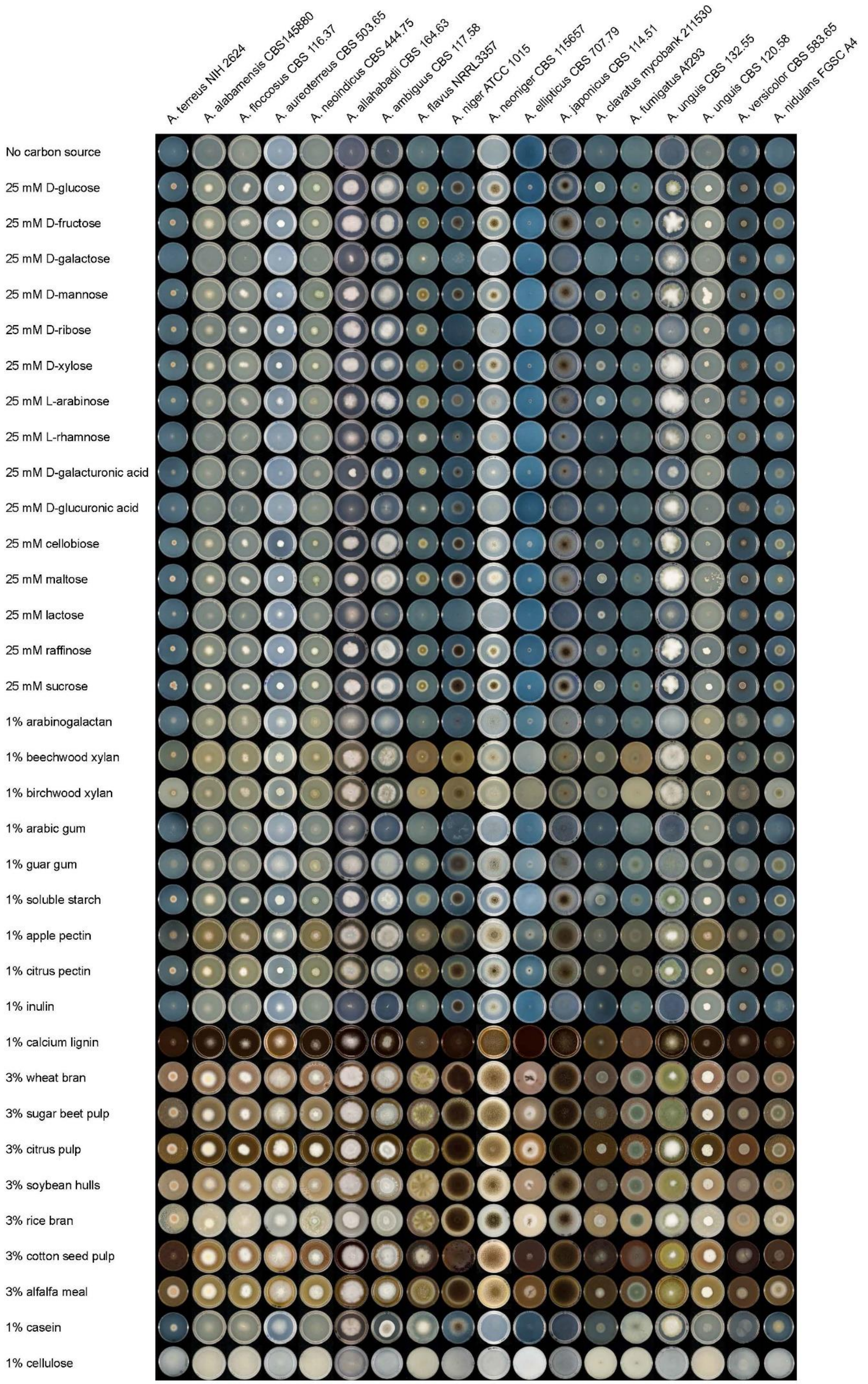


Figure S3. Comparative growth profile of six members of section *Terrei* (*A. terreus, A. alabamensis, A. floccosus, A. aureoterreus, A. neoindicus, A. allahabadii, A. ambiguus*) and 10 other Aspergilli.
